# Oral Lipid Nanoparticles for Improving the Efficiency of Drug Delivery Systems in Ulcerative Colitis: Recent Advances and Future Prospects

**DOI:** 10.3390/pharmaceutics17050547

**Published:** 2025-04-23

**Authors:** Siyu Zhu, Zhenlin Yang, Yulong Liu, Lan Cheng, Dingpei Long, Fangyin Dai

**Affiliations:** 1 State Key Laboratory of Resource Insects, Key Laboratory of Sericultural Biology and Genetic Breeding, Ministry of Agriculture and Rural Affairs, Southwest University, Chongqing 400715, China; zsy3492133451@163.com (S.Z.); yangzl4867@163.com (Z.Y.); liuyi0313@163.com (Y.L.); lancheng@swu.edu.cn (L.C.); 2 College of Sericulture, Textile and Biomass Sciences, Southwest University, Chongqing 400715, China; 3 Yibin Academy of Southwest University, Yibin 644000, China

**Keywords:** lipid nanoparticles, ulcerative colitis, drug delivery efficiency, oral administration, targeted therapy

## Abstract

Ulcerative colitis (UC) is a chronic inflammatory bowel disease characterized by persistent, recurrent, and relapsing inflammation of the mucosal layer. Its pathogenesis is complex and not yet fully understood, with current treatments mainly focused on alleviating symptoms through pharmacological methods. Direct drug administration for UC often leads to poor intestinal bioavailability, suboptimal targeting, and an increased risk of resistance. Therefore, there is an urgent need for effective drug delivery systems. Lipid nanoparticles (LNPs) are promising candidates for UC drug delivery due to their high biocompatibility, stability, and customizable properties. Oral administration, as a preferred treatment approach for UC, offers benefits such as convenience, cost-effectiveness, and better patient compliance. However, oral drug delivery systems must navigate the complex gastrointestinal tract to effectively target colonic lesions, posing significant challenges for LNP-based systems. Researchers are exploring ways to enhance oral delivery efficiency by adjusting LNP composition, surface functionalization, and coating. This article reviews recent advancements in oral LNP research aimed at improving drug delivery efficiency for UC treatment and discusses future prospects.

## 1. Introduction

Inflammatory bowel disease refers to a group of intestinal inflammatory disorders, primarily including ulcerative colitis (UC) and Crohn’s disease. UC is a chronic condition that specifically affects the colon and is characterized by a persistent inflammatory response, though its pathogenesis is still not fully understood. It is generally associated with multiple factors such as defects in the mucosal and epithelial barriers [1,2], immune dysregulation [3], and disturbances in the intestinal microbiota [4]. Clinically, UC manifests as symptoms like bloody diarrhea, with or without mucus, rectal urgency, and varying degrees of abdominal distension and pain [5]. The prevalence of UC is notably higher in European and American countries compared to Asia and Africa. However, with increasing industrialization, the incidence of UC in developing countries is steadily rising, transforming it into a global health concern [6,7].

Pharmacological therapy is currently the primary therapeutic treatment for UC. Traditional anti-inflammatory medications include 5-aminosalicylic acid, which inhibits local inflammatory mediators to reduce intestinal inflammation [8], corticosteroids, which suppress the immune system’s overreaction to reduce the release of inflammatory factors [9], and immunomodulators, which inhibit purine synthesis to reduce the proliferation of T and B lymphocytes and thereby mitigate immune-mediated intestinal damage [10]. These types of medication are usually suitable for mild to moderate patients or for maintenance therapy. Biological treatments are mainly aimed at moderate to severe or refractory cases. Anti-TNF-α drugs reduce inflammation by neutralizing TNF-α and blocking its receptor binding [11,12], anti-integrin drugs prevent immune cell migration by targeting the α4β7 integrin [13], and anti-IL-23 drugs reduce inflammation by inhibiting IL-23 activity and blocking Th17 cell differentiation [14]. Additionally, in recent years, small molecule drugs (such as JAK inhibitors and S1P receptor modulators) have gradually become important treatment options [15]. However, these therapies often face challenges such as instability and poor targeting in the complex biological environment, leading to side effects and limited clinical efficacy. This situation highlights the urgent need for the development of efficient drug delivery systems for UC.

Conventional oral formulations, such as tablets or capsules, are often ineffective in delivering drugs directly to the affected area due to their limited bio-distribution. Advances in nano-biomedical engineering have revealed the potential of nano drug delivery systems (NDDSs) in UC treatment. By exploiting the unique features of the intestinal inflammatory microenvironment, NDDS can create intelligent delivery systems that respond to lesions, using both passive and active targeting strategies to ensure controlled release at the site of inflammation [16,17]. Lipid nanoparticles (LNPs), as a promising type of NDDS, have become a major carrier technology for UC therapy due to their chemical stability, biocompatibility, and potential for surface modification [18]. LNPs effectively protect encapsulated drugs from enzymatic degradation and acidic environments in the gastrointestinal tract, while leveraging the enhanced permeability and retention (EPR) effect and charge-mediated interactions at inflamed intestinal mucosa to improve transmembrane permeability and retention capacity of drugs in lesion areas. Furthermore, active targeting modification strategies (e.g., ligand-receptor-specific recognition) significantly enhance localized drug enrichment at inflammatory sites, minimizing systemic exposure and toxicity risks. By precisely regulating the degradation kinetics of lipid carriers and drug release profiles, LNPs enable sustained-release characteristics of therapeutics, thereby reducing dosing frequency and mitigating the development of drug resistance.

The three main clinical treatment options for UC are oral administration, local delivery, and intravenous administration [19]. Among these, oral delivery stands out due to its non-invasive nature, ease of administration, and cost-effectiveness, making it the preferred choice for long-term treatment adherence. However, oral drug delivery systems using LNPs still face significant technical challenges. These include degradation by gastric acid, metabolism by intestinal enzymes, and the mucus layer’s obstruction, which can lead to non-specific drug release. Additionally, targeting precision to the lesion sites is hindered by the ever-changing conditions within the gastrointestinal tract, and the biosafety assessment for prolonged drug use remains underdeveloped.

Therefore, this paper reviews the strategies and current advancements in oral LNP systems aimed at improving drug delivery for UC, with the goal of providing a theoretical foundation for overcoming existing barriers. In doing so, we hope to encourage further research into oral LNP-based delivery systems for UC, promoting the development of optimized, intelligent, and precise drug delivery systems through interdisciplinary collaboration, ultimately advancing the clinical application of LNPs in UC treatment.

## 2. Oral LNPs Enhance the Delivery Efficiency of Drugs for Treating UC

### 2.1. Challenges of Oral Drug Delivery Systems in Treating UC

Oral LNP delivery systems offer significant advantages in improving drug delivery efficiency for UC treatment by ensuring accurate drug distribution and reducing systemic toxicity. However, the efficiency of LNP-based delivery is significantly impacted by the complex, adaptive barrier system within the gastrointestinal tract. The route of oral drug delivery from the mouth to the colon involves passing through several anatomical regions: the esophagus, stomach, small intestine (duodenum, jejunum, ileum), and large intestine (cecum, colon, rectum). The physiological environments of each region present unique challenges for drug delivery, such as gastric acidity, enzymatic breakdown, and disruption caused by bacterial metabolism, all of which can adversely affect the bioavailability of oral drugs. In designing effective oral drug delivery systems, it is essential to consider the barrier effects of the various parts of the gastrointestinal tract, including both biochemical and physical barriers. These factors must be addressed to ensure the stability of the drug carrier and the precise release of the drug at the inflamed intestinal site. Additionally, chronic UC patients often experience pathological changes in the intestines, such as thinning of the mucus layer, reduced goblet cell activity, and immune cell infiltration, which can interfere with drug absorption kinetics.

#### 2.1.1. Gastrointestinal Barriers to Drug Absorption

Biochemical barriers in the gastrointestinal tract mainly result from fluctuating pH levels and the presence of enzymes. The stomach has an acidic pH that can vary depending on individual differences and food intake, which can affect the stability of acid-sensitive drugs [20]. The gastrointestinal system also contains numerous enzymes, such as pepsin in the stomach and hydrolases, amylase, trypsin, nucleases, and lipases in the intestines. The presence of these enzymes poses challenges for the stability of biopharmaceuticals [21]. Furthermore, the colon’s microbiota significantly influences drug metabolism, which can lead to differences in treatment efficacy and toxicity among patients [22].

The physical barrier system in the intestines plays a crucial role in drug absorption efficiency by providing structural defense and dynamic renewal mechanisms. This system mainly involves the intestinal mucus layer and the epithelial cell barrier. The intestinal mucus layer is a viscoelastic, adhesive gel, with its main component being highly glycosylated mucins, particularly MUC2 [23]. The mucus layer has a bilayered structure, with an inner layer tightly adhered to the intestinal epithelium and a looser outer layer. The barrier function of the outer layer is due to its laminar structure, which consists of dense mucin fibers that effectively block the passage of macromolecules and nanoparticles. Moreover, goblet cells constantly secrete mucus, which shortens the time the medication interacts with the intestinal surface, thus influencing drug retention [24].

The “dynamic barrier” property requires that the drug delivery system has the ability to penetrate the mucus layer or adhere in situ. The intestinal epithelial barrier consists of a monolayer of columnar epithelial cells linked by tight junction proteins, forming a continuous physical barrier that limits drug absorption through cellular bypass while providing a large surface area for transcellular transport [25]. The rapid turnover of the mucus layer and the selective permeability of the epithelial barrier create significant challenges for drug absorption in the intestine.

#### 2.1.2. Impact of UC on Gastrointestinal Physiology

UC is a disease that evolves dynamically, with the degree of colorectal inflammation fluctuating over time, potentially affecting the efficacy of oral medications. The physiological factors influencing the effectiveness of oral drugs in UC include changes in the gut microbiota, pH levels in the colon, and physical barriers. Research shows that UC patients generally have an imbalanced gut flora, with reduced microbial diversity, a decrease in strictly anaerobic bacteria, and a significant increase in facultative anaerobic bacteria balance not only alters the metabolic environment of the gut but also directly impacts the biotransformation and absorption of drugs.

Additionally, the intraluminal pH in the colon of patients with active UC is significantly lowered, approaching gastric levels. This acidic environment can hinder the effectiveness of pH-dependent release agents [26]. Further adds to increased mucus production, immune cell infiltration, and compromised intestinal epithelial junctions, which further affect the absorption of drugs. During UC flare-ups, patients with severe mucosal inflammation may experience increased gastrointestinal motility and diarrhea, which can affect intestinal volume, pH, and mucosal integrity [27,28]. For example, diarrhea shortens the retention time of gastrointestinal tract, which reduces drug absorption. The inflammatory response in the mucosa and severe diarrhea disrupt the resident microbiota in the intestines by altering bacterial composition and diversity, which in turn impacts the secretion of microbial metabolites and impedes UC treatment [29].

In conclusion, active inflammation in UC profoundly impacts gastrointestinal physiology, which in turn affects the efficacy of conventional oral drug delivery systems. Treatment regimens must be tailored according to the disease stage to ensure optimal therapeutic outcomes.

### 2.2. Advantages of Oral LNP in Improving Drug Delivery Efficiency

Recent years have seen extensive investigation into oral NDDSs for treating UC. These systems not only prevent drug degradation in the gastrointestinal tract by encapsulating the drug in nanoscale carriers, but also significantly increase the drug concentration at the lesion site via targeted delivery mechanisms. This optimizes therapeutic effects and minimizes systemic toxicity. LNPs have long been recognized as an ideal carrier for treating various diseases due to their small size, low cost, excellent stability, biocompatibility, and ease of surface modification [30,31,32].

Liposomes (LPs), first proposed by Bangham et al. in 1965 [33], were the first nanocarriers used clinically [33,34]. As nanoscience advanced, the term “LNP” emerged. LPs are primarily composed of lipids and typically operate at the nanoscale, which is why they are considered the earliest form of LNPs [35] LPs are small vesicles made up of lipid molecules, such as phospholipids, arranged in a bilayer structure. The water-filled chamber is located within the lipid bilayer, forming one or more aqueous compartments for drug encapsulation. This bipartite structure allows both hydrophilic and hydrophobic drugs to be encapsulated, facilitating their delivery.

In addition to early LPs, the next generation of lipid nanoparticle carriers primarily includes solid lipid nanoparticles (SLNs) and nanostructured lipid carriers (NLCs). SLNs address the limitations of LPs by improving stability and controlling drug release, while also providing better tolerance and biocompatibility compared to polymer-based nanoparticles [36]. SLNs offer numerous advantages, including easy large-scale manufacturing through high-pressure homogenization, and a solid substrate that effectively prevents drug degradation and aggregation of the carrier. However, SLNs face challenges such as the formation of highly structured crystalline structures during drying, which can compress the drug-loading capacity and cause drug expulsion [37].

NLC, a second-generation lipid nanoparticle system, is derived from solid lipid nanoparticles through component optimization [38]. The key components of NLC include solid lipids, liquid lipids, and surfactants. By using solid-liquid dual-lipid blending technology to create an amorphous matrix system, the drug encapsulation efficiency and controlled release capabilities are significantly enhanced. NLCs possess superior drug loading capacity and release characteristics compared to SLNs, making them a more favorable option for drug delivery systems.

In order to obtain a more thorough comprehension of the distinctions among LPs, SLNs, and NLCs, we prepared Table 1 for a thorough comparison. NLCs are perfect for drug administration with high loading requirements because of their exceptional encapsulation efficiency and stability, which are facilitated by a mixed lipid matrix. On the other hand, SLNs have problems with drug leakage during storage even if they show better encapsulation efficiency. Because of their structural restrictions, LPs have a lower encapsulation efficiency and stability, but their versatility makes them invaluable in several fields.

### 2.3. Main Approaches to Enhancing Oral LNPs for UC Treatment

Enhancing LNP-based oral administration methods for UC treatment focuses on multidimensional technological advancements. The current strategy focuses on overcoming delivery challenges in three areas: carrier engineering (lipid composition, particle size modulation), targeted functional design (physical/biological modifications), and release kinetics optimization (slow, controlled release). The main strategies include increasing acid resistance and improving intestinal tissue cell uptake for better bioavailability, enhancing targeting to UC sites via active and passive targeting. Delivery efficiency is typically improved through various methods, as shown in Table 2, which summarizes recent research progress and the current status of oral drug-loaded LNPs for UC treatment.

#### 2.3.1. Enhancing Drug Bioavailability in Intestinal Tissue

Enhancing drug bioavailability in intestinal tissues via oral LNPs is mainly achieved by improving drug stability and boosting absorption efficiency in the intestines. This involves inhibiting the breakdown of the drug by gastrointestinal enzymes and gastric acid, increasing solubility, and improving its permeability and retention time in the intestine. Previous studies have indicated that the stability of LNPs to lipase is influenced by its lipid components and excipients, with long-chain fatty acids exhibiting higher stability [67]. As listed in Table 2, lipid components such as Soybean phospholipids, Compritol 888 ATO, and Stearic acid possess more stable characteristics due to their long-chain structures. In addition to the selection of lipid components, some adjustments to excipients have also been used to enhance the overall stability of LNPs [45,53]. Conventional liposomes are prone to membrane disruption and premature drug leakage in the gastrointestinal environment. To address this limitation, Zhao et al. proposed an innovative structural modification strategy by replacing cholesterol with tauroursodeoxycholic acid (TUDCA) [45]. As an amphiphilic bile acid derivative, the rigid steroidal ring and hydrophilic sulfonic group of TUDCA significantly enhance the mechanical strength of the lipid bilayer. In vitro simulations demonstrated that TUDCA-incorporated liposomes maintained stable particle size, polydispersity index (PDI), and zeta potential under both pH 1.5 and 6.8 conditions, whereas cholesterol-containing liposomes exhibited significant alterations in PDI and zeta potential. This finding confirms that TUDCA incorporation effectively improves the gastrointestinal stability of liposomal formulations.

Various surface coatings, such as Eudragit S100 [41], chitosan [55], alginate microgels [46], polyelectrolyte complexes [54], and Pluronic F127 [65], have been studied and shown to enhance the overall stability of LNPs. These surface modifications help improve the carrier’s stability under gastrointestinal conditions, contributing to more efficient drug delivery.

The enhanced uptake efficiency in intestinal tissues is primarily due to the increase in the enhanced permeability and retention effect at the colitis site. Optimizing the particle size and surface charge of LNPs can enhance their permeability through intestinal epithelial cells and improve their retention at the colonic site. Typically, during inflammation, positively charged proteins accumulate on the surface of the colonic mucosa [68]. These positively charged proteins can electrostatically interact with negatively charged drug carriers, extending the retention time of the drug at the site of inflammation.

Applying a positive charge to LNPs also facilitates electrostatic adsorption of negatively charged mucin [51] in the intestine, thereby improving the drug’s bioavailability. The physical barrier of the colon can be overcome by polymer coatings, which facilitate the permeation of LNPs [65]. Ma L et al. [65] developed a Pluronic F127-coated mulberry leaf lipid-based nanoparticles, which significantly improved the carrier’s ability to penetrate the mucus layer by reducing the mucus adhesion resistance through a “mucus repulsion-fast penetration” mechanism. This mechanism enabled the nanoparticles to penetrate the intestinal mucus layer more efficiently, thereby improving the drug delivery efficiency in the colon. Pluronic F127 acts as an amphiphilic triblock copolymer with hydrophilic polyethylene oxide chain segments that form a dense hydration layer on the surface of the nanoparticles, which significantly reduces hydrophobic interactions with intestinal mucus glycoproteins such as MUC2.

#### 2.3.2. Optimizing Drug Targeting to the UC Site

UC drug targeting is enhanced through two primary strategies: passive targeting and active targeting.

##### Passive Targeting

Passive intestinal targeting primarily relies on the characteristics of the ulcerative colitis lesion site and the physicochemical properties of the drug carrier, promoting the natural accumulation of the drug in the colon. This is mainly achieved by modifying the surface coatings of LNPs and adjusting the particle size to target the inflamed areas of the colon. Specific pH-, enzyme-, and charge-sensitive polymers are used to encapsulate LNPs for oral administration of ulcerative colitis medications. For example, Eudragit S100 is a polymer that remains stable in acidic environments and dissolves in alkaline conditions, making it suitable for targeted release in the intestines [41,61]. A study by Aib S et al. on Eudragit S100-coated LPs revealed that uncoated LPs exhibited burst release in both pH 1.2 and pH 7.4 media, with no significant pH-dependent release modulation [41]. Furthermore, uncoated LPs underwent severe degradation in gastric conditions, resulting in substantial drug loss and minimal drug accumulation in the colon. In contrast, coated LPs demonstrated pH-responsive release behavior: drug leakage was negligible (<5%) at pH 1.2, while a marked increase in drug release (>80%) occurred at pH 7.4. This pH-sensitive design significantly enhanced colon-targeted delivery efficiency by minimizing premature drug loss and ensuring site-specific release.

Chitosan, a naturally occurring cationic polysaccharide, exhibits protonation in acidic conditions, giving it a positive charge that facilitates interaction with mucin. This helps maintain stability in the stomach, after which chitosan is broken down by enzymes produced by colonic bacteria in the intestine [69]. Additionally, LNPs with covalent enzyme-sensitive linkages provide an effective enzyme-responsive targeted delivery strategy, improving the drug’s precision and retention [70]. S Chen et al. [70] developed chitosan-modified LNPs for precise UC treatment through enzyme-responsive release and charge-targeting mechanisms, effectively addressing the non-specific release and low therapeutic efficacy of conventional oral drugs in the gastrointestinal tract. This design leveraged the cationic properties of chitosan to enhance electrostatic adsorption between nanoparticles and negatively charged inflamed intestinal mucosa, thereby prolonging local drug retention. Furthermore, by incorporating ester bonds as linkers, which are specifically cleaved by esterases highly expressed in the colon, the system achieved targeted drug release at lesion sites. This strategy increased drug enrichment in inflamed regions while minimizing gastrointestinal loss, significantly enhancing therapeutic outcomes.

##### Active Targeting

Active targeting of UC is mainly mediated by ligand-receptor interactions. By attaching ligands to the surface of LNPs, this strategy utilizes surface receptors on inflammatory cells in UC, allowing for more specific targeting of inflamed colonic regions [71]. For example, folic acid, a water-soluble vitamin, binds highly specifically to folate receptors, which are overexpressed on various cancer cells and inflammatory cells, including damaged colonic epithelial cells and activated immune cells [72]. Yang S et al. developed a folate-modified chicoric acid liposome that leverages the high expression of folate receptors on activated macrophages to achieve precise targeting of inflammatory colonic macrophages. This nanotherapeutic system significantly enhances therapeutic efficacy while minimizing systemic toxicity in UC by modulating macrophage polarization (suppressing pro-inflammatory M1 phenotype and promoting anti-inflammatory M2 phenotype) and inhibiting the Toll-like receptor 4/nuclear factor kappa-B (TLR4/NF-κB) inflammatory signaling pathway. The dual-action mechanism combines targeted delivery with immunoregulation, demonstrating robust anti-inflammatory effects and tissue repair promotion in preclinical UC models [47].

To enhance the stability of LNPs surface ligands in the gastrointestinal tract, surface-coated LNPs are also being investigated for oral drug delivery in UC treatment. In addition to folate receptor targeting, LNPs utilizing other inflammatory cell receptors, such as phosphatidylserine [46] and glycosylation modifications [65], have been used in preclinical studies for delivering UC drugs.

## 3. Application of Oral LNPs in Enhancing Drug Delivery Efficiency for UC Treatment

Numerous preclinical studies have focused on optimizing LNPs drug delivery methods to accelerate the clinical progression of oral LNPs treatment for UC. These studies emphasize the optimization of lipids, surfactants, and excipients, as well as the formulation of novel lipid nanoparticle medications to enhance therapeutic efficacy in UC. Ginger-derived lipid carriers, nanoparticles extracted from edible ginger, have shown promise in the exploration of oral nucleic acids and compounds for UC treatment, demonstrating high delivery efficiency and safety [62,64].

In addition to ginger-derived lipid carriers, various naturally available lipids from sources such as mulberry [65] and tea leaves [66] have been formulated into LNPs for oral drug administration. Researchers have also developed innovative liposome nanoparticles using unique lipid molecules to replicate the primary components and ratios of natural lipids [73]. This has helped to improve the chemical consistency of plant-derived lipids and reduce batch-to-batch variability.

Specific binary solid lipid nanoparticles have been studied for oral administration of UC medications by optimizing lipid structure and composition. For example, Sharma et al. utilized fatty acids and triglycerides to create binary solid lipid nanoparticles, which exhibited good encapsulation efficiency and controlled drug release for the oral delivery of curcumin in UC treatment [52]. Moreover, polymer-lipid hybrid nanoparticles (PLHNs) have recently attracted significant interest as an innovative drug delivery strategy. PLHNs aim to overcome the limitations of polymer nanoparticles or liposomes alone, improving both drug delivery efficacy and safety, and functioning as a co-delivery system for two molecules [74,75]. A research team used lipid-polymer hybrid nanoparticles (R8-PEG-PPNPs) for oral delivery of superoxide dismutase to treat UC [75]. These nanoparticles have good stability and biocompatibility, effectively protecting superoxide dismutase from gastrointestinal degradation and precisely delivering it to the colon. Experimental results indicate that this delivery method significantly improves the oral delivery efficiency of superoxide dismutase and its therapeutic effect on ulcerative colitis, opening new avenues for the precise treatment of oxidative stress-related intestinal diseases.

LNPs are currently employed for the oral administration of conventional UC medications, such as 5-aminosalicylic acid, immunosuppressants, and corticosteroids, with preclinical studies validating their effectiveness. These studies show that LNPs enhance drug delivery efficiency and improve therapeutic outcomes. In addition, some potential candidate UC drugs, including other anti-inflammatory drugs [55,60], plant-derived drugs [49,57,58,59,63], and nucleic acid therapies [62,73], have been explored using oral LNP delivery systems.

Combination therapy, which utilizes multiple drugs, has also been reported as an effective method for treating UC. For example, Dianzani et al. studied the synergistic effects of dexamethasone and butyrate in UC treatment [50]. Their research demonstrated that combining these two agents can reduce side effects and effectively alleviate UC symptoms.

The development of UC animal models, particularly the interleukin-10 knockout mouse model, plays a critical role in understanding UC’s pathogenesis and evaluating therapeutic agents. The mouse model accurately mimics chronic inflammation, offering valuable insights for preclinical drug testing. DSS-induced UC mouse models are also widely used to simulate human UC’s immunological and histopathological features, providing a platform for studying the stability and delivery effectiveness of oral LNP systems. These animal models are essential for advancing the understanding of UC and optimizing treatment strategies using oral LNP-based drug delivery systems.

LNPs have demonstrated significant clinical efficacy in treating various diseases within the field of nanomedicine. Initially described in 1965, LPs were FDA-approved in the 1990s for the use of doxorubicin in the treatment of breast cancer, ovarian cancer, and other solid tumors. In recent years, the development of gene therapy has significantly increased the use of LNPs for nucleic acid drug delivery [76,77]. Since the approval of the first LNP drug delivery system, significant progress has been made in various fields such as oncology, infectious diseases, and gene therapy [78,79]. However, LNPs are in an early clinical phase for UC treatment. While they have shown promise, there are several challenges in their clinical application. Further optimization in areas such as biocompatibility, stability, targeting, preparation techniques, drug release control, and cost-effectiveness is needed to enhance the clinical use of LNPs in UC treatment.

## 4. Challenges and Future of Oral LNP-Based Drug Delivery Systems for UC Treatment

### 4.1. Challenges in Oral LNP Preparation Technology

LNPs preparation techniques have been extensively studied in various contexts [80,81]. This section provides a concise overview of the LNPs preparation methods currently employed for oral drug delivery in UC treatment. Among these, the Thin-Film method is frequently used to generate liposomes and natural lipid nanoparticles. While the film hydration method is simple and works well for lipophilic drug encapsulation, it poses some limitations, particularly regarding the stability of the resulting liposomes.

Other methods, such as the reverse phase evaporation and solvent injection methods, also have comparable limitations in terms of efficiency and stability. The hot homogenization technique is widely used for preparing SLNs and NLCs [82]. This method effectively reduces the particle size, enhances the uniformity of particle size distribution, and improves drug encapsulation efficiency. However, the high temperatures required for this process make it unsuitable for heat-sensitive drugs.

Cold homogenization, while effective, presents challenges with drug solubility and requires strict operational conditions [82]. Microemulsion technology requires a larger quantity of surfactants, which could increase the toxicity of the formulation and complicate the preparation process [83]. Despite the various advantages, each of these methods has its own limitations, and the selection of the most suitable preparation technology depends on the properties of the drug and the formulation requirements.

To address these technical challenges, future efforts should focus on integrating AI-driven parameter optimization models. These models can help overcome existing bottlenecks in preparation technology and accelerate the clinical translation of LNP formulations for UC treatment. In summary, the future of oral LNP preparation methods for UC treatment will require careful optimization of drug characteristics, process adaptation, and alignment with clinical needs.

### 4.2. Development Trends and Future Directions in Oral LNP Treatment for UC

Considering the complex nature of UC’s pathological mechanisms and the progress in preclinical research, future clinical developments in oral LNP therapy will focus on two key areas: the creation of intelligent, responsive delivery systems and the integration of interdisciplinary technologies. A comprehensive translational medicine system, integrating research, development, production, and regulation, must be established simultaneously to ensure the successful clinical implementation of oral LNPs.

UC treatment is shifting from traditional non-targeted therapies to more advanced targeted therapies. This trend reflects a deeper understanding of disease mechanisms and the refinement of treatment strategies. Smart LNP delivery technology combines environmental responsiveness with targeted therapy, enabling controlled drug release in response to changes in the intestinal microenvironment, such as pH, charge, enzymes, and inflammatory factors. Moreover, LNP-based gene therapies are capable of delivering therapeutic nucleic acids, like mRNA or siRNA, to targeted areas, modulating the signaling pathways involved in UC development.

Interdisciplinary research has led to innovative concepts for improving the LNP delivery system, with a focus on two main areas: integrating artificial intelligence (AI) with data analysis and exploring the interaction between the gut microbiome and drug delivery systems. AI-driven rational design enables the optimization of LNP composition and structure, making the process more efficient and cost-effective [84]. Furthermore, AI has been applied to lipid screening, helping to streamline lipid development and improve bioavailability in LNP formulations [85].

Recent studies have increasingly highlighted the significant role of the gut microbiome in UC pathogenesis and its synergy with drug delivery systems. The LNP delivery system can indirectly regulate the balance of gut microbiota by modulating the active components of the microbiota, thereby enhancing the efficacy of drugs in diseases such as inflammatory bowel disease. For instance, drugs delivered to the colon via LNP have been shown to interact with the gut flora and modify the intestinal microbiota composition, helping treat UC [86]. Additionally, in vitro culturing of gut microbiota combined with LNP-delivered anti-inflammatory drugs has demonstrated the potential to prevent chronic inflammation in interleukin-10 knockout mice [64].

### 4.3. Economic and Regulatory Challenges in Oral LNP Treatment for UC

Despite advancements in technology, oral LNP treatment for UC remains in the preclinical research phase. Several challenges need to be addressed, including improving cost-effectiveness evaluation and optimizing the regulatory approval process. Cost-effectiveness analysis is essential for clinical translation, assessing the economic feasibility of new technologies by considering R&D costs, production expenses, and treatment outcomes [87].

The production of LNPs remains a significant cost challenge, primarily due to complex preparation processes and the need for high-quality raw materials. Technological advancements and large-scale production are expected to reduce costs, enabling broader clinical application [88,89,90]. Additionally, the economic impact of LNP treatment for UC requires long-term cost-effectiveness evaluation compared to current therapeutic options, such as traditional pharmacological treatments.

Refining regulatory standards and approval processes is crucial for facilitating the clinical translation of LNP treatment for UC. Regulatory agencies are actively updating and improving the approval process to accommodate novel technologies, accelerating the clinical implementation of LNP-based therapies [91,92,93].

## 5. Conclusions

Oral LNP-based drug delivery systems represent a promising approach to UC treatment, offering the potential to transition from symptom relief to lesion repair through their innovative design and interdisciplinary integration. Despite the progress made in preclinical research, the clinical application of oral LNPs for UC remains in its early stages, with several challenges yet to be addressed. These include improving stability, controlled release, and targeting, as well as overcoming technical limitations in preparation methods. Innovations in smart LNP delivery, such as pH-responsive, enzyme-sensitive, and electrostatic adsorption strategies, have been developed to enhance site-specific drug release. However, the degradation risks of LNPs in gastric acid and enzymatic environments, and the need to balance mucus clearance with epithelial cell permeation, require further optimization. Moreover, clinical trial design and regulatory frameworks need to be refined to ensure the safe and efficient translation of these systems into practice. Cost-effectiveness analysis and large-scale production are crucial to making LNP-based therapies commercially viable. Future research must align basic innovations with clinical needs, optimizing LNP stability, targeting, and preparation techniques while focusing on improving regulatory standards to facilitate the successful clinical application of LNP-based therapies for UC.

## Figures and Tables

**Table 1:** Comparative differences among LPs, SLNs, and NLCs.

Feature	LPs	SLNs	NLCs
Structure	Phospholipid bilayer composition with an internal aqueous core	Consists of a single solid lipid matrix that forms a dense crystalline structure.	Mixing of solid and liquid lipids to form a non-perfect crystalline structure.
Drug Encapsulation	High encapsulation efficiency for hydrophilic drugs, low encapsulation efficiency for fat-soluble drugs.	Suitable for fat-soluble drugs, encapsulation is less efficient due to the solid matrix.	Suitable for fat-soluble drugs, encapsulation efficiency is higher than SLN due to the presence of liquid oil.
Drug release behavior	Rapid initial burst release (due to membrane permeability) or sustained release (modified via surface functionalization).	Release is sustained due to solid lipid matrix, release rate can be controlled by lipid type and surfactant.	Similar to SLN, sustained release, but initial release may be faster due to the presence of liquid oils.
Application scenario	Vaccines, gene delivery, combination therapy requiring double loading	Hydrophobic drugs requiring slow release	Hydrophobic drugs with high drug loading requirements requiring long term stability for formulation development.
Storage stability	Prone to oxidation/hydrolysis, requires cryoprotectants for long-term storage.	High physical stability but may undergo lipid recrystallization over time.	Improved stability due to reduced recrystallization, liquid lipids prevent drug leakage.

**Table 2:** Current status of preclinical research on oral LNP for improving the treatment efficiency of UC.

Type of LNPs	Year of Publication	Preparation	Lipid Composition	LNPs-Loaded Drugs	Main Effect	Ref.
LPs	2021	Solvent Injection	Lecithin, cholesterol	Curcumin	Improved drug stability in acidic environments (22% drug release at pH 1.2 vs. 76% in control)	[39]
LPs	2021	Thin-Film Hydration	Egg yolk lecithin, cholesterol	Celastrol	The pectin-trimethyl chitosan wrapped LPs improved stability and intestinal adhesion (mucin adhesion in the experimental group was about 1.8 times higher than in the blank group)	[40]
LPs	2022	Thin-Film Hydration	Soybean phospho-lipid, cholesterol	Mesalazine, Curcumin	Eudragit S-100 wrapped LPs to achieve passive pH targeting (8% drug release at pH 1.2 and 88% at pH 7.4)	[41]
LPs	2023	Thin-Film Hydration	Soybean, cholesterol phospholipid	Curcumin	Folic acid-modified LPs enable active targeting (macrophage uptake several times higher than in the blank group), Pectin chitosan hydrogel encapsulation of LPs to improve stability in the stomach and small intestine (less than 10% drug release)	[42]
LPs	2023	Reverse-Phase Evaporation	Soy lecithin, cholesterol	Superoxide Dismutase	Improved enzyme drug stability (27% retention of enzyme activity at low pH and strong digestive enzymes vs. 1% in control)	[43]
LPs	2023	Solvent Injection	Lecithin, cholesterol	Budesonide	The drug is linked to linoleate bonds to LPs to improve acidic stability and enable enzyme response targeting. Total of 5% release at pH 1.5, rapid release in the presence of esterase, no release in the absence of the enzyme.	[44]
LPs	2024	Thin-Film Hydration	Soybean phospholipids	Emodin	Replacement of cholesterol by taurine deoxycholic acid enhances the stability of LPs in acidic environments and mucus layer penetration (3-fold higher penetration rate than normal liposomes)	[45]
LPs	2023	Thin-Film Dispersion	Phosphatidylcholine, cholesterol	Genistein	The alginate microgels encapsulated LPs to enhance stability and pH targeting, with 8% release at pH 1.2 (control 60%) and 87% release at pH 6.8 (control 39%).	[46]
LPs	2024	Double Emulsion Ultrasound	Egg yolk phospholipid, cholesterol	Chicory acid	Folic acid modification of LPs improves active targeting ability	[47]
LPs	2024	Thin-Film Dispersion	Soybean lecithin, cholesterol	Curcumin, Probiotic	Chitosan blends encapsulating LPs elevated probiotic survival and intestinal adhesion in the gastrointestinal environment, with a 10-fold higher survival rate than the LP group.	[48]
LPs	2024	Thin-Film Dispersion	Soybean lecithin, cholesterol	Chlorogenic acid	Folate-TPGS modification of LPs to improve active targeting	[49]
SLNs	2017	Warm Microemulsion	Epikuron 200, Soya Lecithin	Dexamethasone, Cholesteryl butyrate	Improving the stability of Dexamethasone and Cholesteryl bu-tyrate during synergistic drug treatment	[50]
SLNs		Hot Homogenization	Compritol, Phospholipon 90 G	Budesonide	Polyethyleneimine enhances intestinal retention by cationizing SLNs, and Eudragit S100 encapsulation confers pH-passive targeting and stability, with 16% drug release at pH 1.2 versus 60% in the blank group	[51]
SLNs	2018	Emulsification Solvent Evaporation	Tristearins, Tearic acid, Soya Lecithin	Curcumin	Use of dibasic lipids (stearic acid and tristearin) to improve drug gastrointestinal stability and storage stability	[52]
SLNs	2019	Solvent Emulsification	stearic acid, Lecithin	Eluxadoline	Optimizing lipid composition to improve SLNs stability	[53]
SLNs	2020	Hot Melting Ultrasonication	Compritol 888 ATO	Budesonide	Modulation of the number of layers of polyelectrolyte complexes encapsulating SLNs enhances stability and enzyme response targeting. 14% release at pH 1.2 (control 57%), 75% release under cellulase conditions, 30% without enzyme.	[54]
SLNs	2023	Hot Homogenization	Cetyl palmitate	Fexofenadine	By optimizing the molecular weight of chitosan and combining it with SLNs encapsulation, the storage and release stability and adhesion of the drug were enhanced.	[55]
NLCs	2024	Hot Homogenization	Precirol ATO 5, Miglyol 812	Budesonide	Increased solubility and retention of drugs in the intestinal tract	[56]
NLCs	2013	Hot Emulsification	Precirol ATO 5, Miglyol 812N/F	Curcumin	Enhanced colonic retention rather than permeability, with permeability 30-fold lower than control.	[57]
NLCs	2016	Hot Melt Emulsification	Precirol ATO 5, Olive oil	Oleuropein	Improve drug stability and intestinal mucosal aggregation	[58]
NLCs	2020	Hot Homogenization	Compritol 888 ATO, Olive oil	Berberine	Improved drug stability and intestinal solubility in an acidic environment. Total of 11% drug release at pH 1.2 (39% in the blank group), higher uptake by Caco-2 cells than in the blank group	[59]
NLCs	2020	Hot Emulsification	Glyceryl monostearate, Geraniol	Celecoxib	Eudragit S100 wrapped NLCs to achieve pH passive targeting. >1% drug release at pH 2 and 90% at pH 7.4	[60]
NLCs	2020	Microemulsion Technology	Compritol 888 ATO, Oleic acid	Tacrolimus	Cetyltrimethyl ammonium bromide was added as a surfactant to improve adhesion, Eudragit FS100 wrapped NLCs to achieve pH passive targeting and long-lasting sustained release. drug release rates of 13% at pH 1.2, 26% at pH 4.6, and more than 60% at pH 7.4 were achieved, allowing for controlled sustained release for up to 72 h	[61]
Natural-LNP	2024	Thin-Film Dispersion	Ginger derived lipids	siRNA	Biocompatibility is higher than the commercially available liposomal formulation DC-Chol/DOPE	[62]
Natural-LNP	2017	Thin-Film Dispersion	Ginger derived lipids	6-shogaol	Improvement of drug stability in acidic environment with less than 20% drug release at pH 1.2 compared to more than 60% in the blank group	[63]
Natural-LNP	2020	Thin-Film Dispersion	Ginger derived lipids	M13	Enhancement of intestinal microbial drug uptake and modification of the composition of the inflamed intestinal microbiota and its secreted metabolites in isolated cultures	[64]
Natural-LNP	2022	Thin-Film Hydration	Mulberry leaf derived lipids	CRISPR/Cas9	Pluronic F127 encapsulation enhances stability and intestinal permeability, the galactose-terminal moiety of LNP enhances active targeting, and macrophage uptake efficiency exceeds that of the control by seven-fold	[65]
Natural-LNP	2024	Thin-Film Hydration	Tea derived lipids	Curcumin	Reduce the risk of spreading harmful bacteria and increase the proportion of probiotic gut bacteria	[66]

Comment: 1. Natural-LNP (Natural Lipid Nanoparticles): Typically composed of lipids from natural sources (such as plants), they possess excellent biocompatibility, low toxicity, and biodegradability, making them suitable for in vivo applications. 2. Details of the loaded medication: Curcumin (a natural polyphenolic compound derived from turmeric), Mesalazine (5-ASA), Celastrol (a natural triterpenoid compound derived from Thunder God Vine), Superoxide Dismutase (enzyme biopreparation), Emodin (a natural anthraquinone compound, derived from plants such as rhubarb), Budesonide (glucocorticoid), Genistein (a natural isoflavone derived from soybeans and other leguminous plants), Chicory acid (a natural phenolic compound derived from chicory), Probiotic (Beneficial active microorganisms for the human body), Chlorogenic acid (a natural phenolic compound, derived from honeysuckle and other plants), Eluxadoline (a drug used to treat irritable bowel syndrome), Fexofenadine (antihistamine), Dexamethasone (glucocorticoid), Cholesteryl butyrate (chemically modified lipid), Tacrolimus (immunosuppressant), Berberine (a natural alkaloid derived from plants such as Coptis chinensis), Celecoxib (non-steroidal anti-inflammatory drug), 6-Shogaol (a natural phenolic compound derived from turmeric), M13 (glutathione-conjugated, 6-shogaol’s phase-II metabolite), siRNA (nucleic acid drug), and CRISPR/Cas9 (gene editing tool).

## Data Availability

No new data were created or analyzed in this study. All relevant findings are based on theoretical analysis/cited literature.
